# MicroRNA-4651 represses hepatocellular carcinoma cell growth and facilitates apoptosis via targeting FOXP4

**DOI:** 10.1042/BSR20194011

**Published:** 2020-06-10

**Authors:** Yun Li, Xiaoying Wang, Zhonggang Li, Bingxia Liu, Chaoyu Wu

**Affiliations:** 1 Infectious Disease Department, Linyi Central Hospital, Shandong Province, China, 276400; 2 Neurosurgery Department, Linyi People’s Hospital, Shandong Province, China, 276400; 3 Respiratory Medicine Department, Linyi Central Hospital, Shandong Province, China, 276400

**Keywords:** apoptosis, FOXP4, hepatocellular carcinoma, miR-4651, proliferation

## Abstract

MicroRNAs (miRNAs) belong to the subgroup of small noncoding RNAs, which typically serve as important gene regulators to participate in different biological events, such as tumor cell growth and apoptosis. Recent studies indicated microRNA-4651 (miR-4651) was involved in hepatocellular carcinoma (HCC) progression. The certain role of miRNA-4651 during the progression of HCC, however, remains unclear. Herein, we investigated the mRNA expression level of miR-4651 in HCC tissues and HCC cell lines and found miR-4651 was noticeably down-regulated compared with the normal liver tissues and QSG-7701 cell line, respectively. Then, miR-4561 overexpression obviously repressed the proliferation and promoted apoptosis in two HCC cell lines. Interestingly, we further identified that miR-4561 could directly interact with FOXP4 in HCC cells by using bio-informatic method and report assay. Moreover, forced expression of FOXP4 showed an opposite effect compared with miR-4561 in HCC cell lines. Hence, our findings strongly indicated that miR-4561 regulated the HCC cell growth and apoptosis mainly through targeting the FOXP4 genes. Clinically, the miR-4561/FOXP4 axis might be a potential target for therapeutic application of HCC patient treatment.

## Introduction

Hepatocellular carcinoma (HCC) ranks fifth among the malignant tumors in morbidity and the third in leading cause of cancer-related deaths worldwide [2,7,16,15]. HCC tumorigenesis process has a complicated mechanism and relates to many gene alterations, such as microRNAs (miRNAs) [6,14,9,17]. Although numerous investigations evidenced many signal pathways are involved in HCC cell proliferation and apoptosis, the molecular mechanism underlying cell proliferation and apoptosis of HHC is still calling for new breakthrough.

MicroRNAs (miRNAs), a subgroup of small non-coding single-stranded RNAs, generally contain 18–24 nucleotides [1,5]. Functionally, miRNAs typically silence their mRNA target through binding to complementary recognition sequences of mRNA and inhibiting its translation [22,12]. The miRNAs, serve as gene regulators, have been proved to take part in multiple biological events, such as cell proliferation, differentiation and tumorigenesis [3,22,12]. MiR-4651, encoded by the miRNA-4651 gene, is located at the 75915197th to 75915269th base of chr7 [20,23]. Previous studies confirmed miRNA-4651 was expressed in patients with aflatoxin B1-positive HCC, and might serve as a potential biomarker for HCC prognosis [23], which strongly indicated that miRNA-4651 might be participating in HCC progression. Laterally, miRNA-4651 has been identified to regulate nonsense-mediated mRNA decay through interacting with SMG9 mRNA [13]. However, the certain role and mechanism of miRNA-4651 during the progression of HCC need to be further elucidated.

Here, our study aimed to demonstrate the exact role of miR-4651 in HCC pathogenesis. And we found a dramatically decreased in miR-4651 expression in HCC cells. Forced expression of miR-4651 led to inhibit HCC cell growth and promote apoptosis in HCC cell lines. Furthermore, we investigated interaction between miR-4561 and forkead box P4 (FOXP4), and authenticated miR-4561 regulated the HCC cell growth and apoptosis mainly by interacting with the FOXP4. In conclusion, we confirmed that miR-4561 repressed cell growth and apoptosis of HCC through regulating its target gene, FOXP4.

## Materials and methods

### Tissue collection

The HCC tissues and adjacent normal liver tissues were collected from Linyi Central Hospital between 2010 and 2017. Thirty pairs of tissues in total were analyzed in the present study. No systemic treatment of chemotherapy or radiotherapy was conducted in these patients before surgery. All of patients had got the written informed consent before surgery. The study followed the ethics committee of Linyi Central Hospital guidance. We maintained all specimens at −80°C until use.

### Cell culture

We cultured HCC cell lines HepG2 (TCHu-72) in minimum essential medium (MEM) (Gibco, 41500034). Meanwhile, HCC cell lines HuH-7 (SCSP-526) were cultured in Dulbecco’s modified Eagle Medium (DMEM) (Invitrogen, 11960-044) with 1% Glutamax (Invitrogen, 35050-061), 1% non-essential amino acids, 100× (Invitrogen, 11140-050). HCC cell lines SNU-387 (SCSP-5046) were cultured in RPMI-1640 Medium (Invitrogen, 11875-093) with 1% Glutamax (Invitrogen, 35050-061), 1% sodium pyruvate 100 mM Solution (Invitrogen 11360070). HCC cell lines Li-7 (TCHu-183) and SMMC-7721 (TCHu-52) were cultured in RPMI-1640 Medium (GIBCO, 31800022). Normal liver cell lines QSG-7701 (GNHu-7) were cultured in RPMI-1640 Medium (GIBCO, 31800022). All media were added with 10% fetal bovine serum (FBS), respectively. Humidified atmosphere containing 5% CO_2_ at 37°C was performed to incubate the cell lines mentioned above. We purchased the cell lines from the Institute of Biochemistry and Cell Biology at the Chinese Academy of Science (Shanghai, China).

### Plasmid and transfection

The miR-4651-mimics, negative control, FOXP4 overexpression (OE-FOXP4) and negative overexpression control (OE-control) were supplied by GenePharma (Shanghai, China). According to the manufacturer’s instructions, we transfected the plasmids into HepG2 and SNU-387 cells using Lipofectamine 2000 Transfection Reagent (Invitrogen).

### Quantitative real-time PCR assay

We extracted total RNAs from tissues or cultured cells using TRIzol reagent (Invitrogen, Carlsbad, CA), following the manufacturer’s instructions. For quantitative real-time PCR (qRT-PCR), we used a reverse transcription kit (Takara, Dalian, China) to reverse transcribed total RNA into cDNA according to the manufacturer’s protocol; while we used a stem–loop RT-qPCR method to generate miRNAs. Finally, we conducted qRT-PCR in ABI StepOnePlus™ real-time PCR system (Applied Biosystems, Foster City, U.S.A.). For miRNA and mRNA expression detection, U6 and GAPDH were applied as internal controls, respectively. The gene-specific primers are used as shown in Table 1.

**Table 1:** Gene-specific primers used for qRT-PCR

Gene	Primer	Sequence 5′ to 3′
*miR-4651*	Forward	ACACTCCAGCTGGGCGGGGUGGGUGAGG
	Reverse	CTCAACTGGTGTCGTGGAGTCGGCAATTCAGTTGAGGCCCGACC
*FOXP4*	Forward	ATCGGCAGCTGACGCTAAATGAGA
	Reverse	AAACACTTGTGCAGGCTGAGGTTG
*U6*	RT	CGCTTCACGAATTTGCGTGTCAT
	Forward	CTCGCTTCGGCAGCACA
	Reverse	AACGCTTCACGAATTTGCGT
*GAPDH*	Forward	ACACCCACTCCTCCACCTTT
	Reverse	TTACTCCTTGGAGGCCATGT

### Cell counting Kit-8 assay

Cell Counting Kit-8 (CCK-8) assay was used to detect cell growth of HepG2 and SNU-387. Each group was incubated with a density of 10^4^ cells in 96-well plates. Cells in each well was incubated lasted for 2 h at day 0, 1, 2, 3 and 4 with CCK-8 reagent (Doindo, Japan). We measured the optical density at 450 nm using an automatic microplate reader (Synergy4; BioTek).

### Flow cytometric assay

For apoptosis detection, the Hep G2 and SNU-387 cells were transfected with miR-4651 mimics, negative control, miR-4651 mimics+OE-FOXP4, negative control+OE-FOXP4, miR-4651 mimics+OE-control, negative control+OE-control, respectively. The cells were collected at 24 h after transfection. Then, we used an Annexin V-FITC/PI apoptosis detection kit (Invitrogen) to label the HepG2 and SNU-387 with Annexin V and PI. Flow cytometry (FACScan; BD Biosciences) was used to detect and analyze the fluorescence (FL1) and red fluorescence (FL2).

### Target predication

The predicated target of miR-4651 were obtained from miRDB (http://www.mirdb.org/).

### Dual luciferase reporter assay

We constructed pGL3-promoter drived wild-type or mutant FOXP4 luciferase reporter with the binding site for miR-4651 in them. And then, we used Lipofectamine 2000 (Invitrogen) to transfect the luciferase reporter with miR-4651 negative control or miR-4651 mimics into the HepG2 and SNU-387 cells. The firefly luciferase activity was deteccted at 48 h after transfection using Dual Luciferase Reporter Assay system (Promega).

### Western blot assay

For protein isolation after treatments, cells were lysed in the RIPA buffer (Beyotime, China). And then, we quantified the whole proteins and boiled in sodium dodecyl sulfate. The SDS-PAGE gel and nitrocellulose membranes (GE Healthcare) were utilized to separate the proteins and be transferred onto by separated proteins, respectively. We used primary antibodies to incubate the membranes overnight at 4°C. After washing the membranes for five times by phosphate-buffered saline supplemented with Tween 20 (PBST), the corresponding horseradish peroxidase-conjugated secondary antibodies (Santa Cruz) were used to incubate the membranes for 1 h at room temperature. The Super Signal West Femto kit (Pierce, Rockford, IL) was utilized to bring the bands on the membranes into visualization in the final. The primary antibodies and secondary antibody were used as following: rabbit anti-FOXP4 (1:1000, Abcam, ab17726), rabbit anti-GAPDH (1:5000, Abcam, ab181602) and IRdye 800-conjugated goat anti-rabbit IgG. We used GAPDH as the endogenous control in this assay.

### Statistical analysis

For significant difference analysis, we performed all the data using GraphPad Prism 5.0 software. All data were analyzed using the two-tailed Student’s *t*-test and shown as the mean ± SD. *P<0.05, **P<0.01, *** *P*<0.001.

## Results

### Expression of miR-4651 had a down-regulation in HCCcells

**Figure 1:**
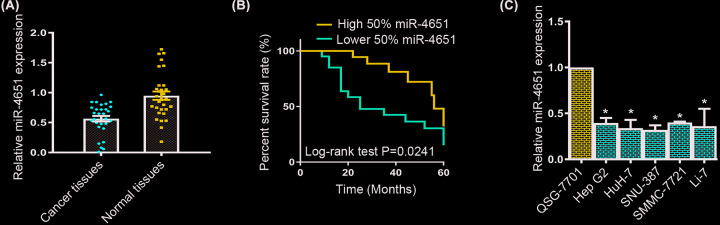
Expression of miR-4651 was decreased in HCC tissues and HCC cell lines (**A**) qRT-PCR assay showed a down-regulation of miR-4651 expression in HCC tissues compared with adjacent normal tissues (*n*=30). (**B**) Overall survival of HCC patients with different miR-4651 expression was measured by Kaplan–Meier analysis (*n*=30, *P*=0.0241). (**C**) The expression of miR-4651 was decreased in HCC cells HepG2, HuH-7, SNU-387, SMMC-7721 and Li-7 cells compared with QSG-7701 cells by qRT-PCR analysis. * in represented the significantly change between HCC cell lines and QSG-7701 cell line.

To investigate the function of miR-4651 during HCC development, we first evaluated the expression of miR-4651 in HCC tissues and adjacent normal liver tissues. Compared with the matched normal tissues, we observed the miR-4651 expression was obviously down-regulated in the HCC tissues by qRT-PCR analysis (Figure 1A). Then, we divided the patients into two cohorts by medium cut-off of normalized miR-4651 expression in HCC tissues. Kaplan–Meier survival curve was conducted to display that patients with low 50% miR-4651 expression rather than high 50% miR-4651 expression suffered worse HCC prognosis (Figure 1B). Furthermore, we also detected miR-4651 expression in HCC cells HepG2, HuH-7, SNU-387, SMMC-7721 and Li-7. Consistent with HCC tissues, down-regulation of miR-4651 expression was also observed in HCC cell lines rather than normal liver cells QSG-7701(Figure 1C). Therefore, decreased miR-4651 expression in both HCC tissues and HCC cell lines indicated miR-4651 might take an essential role in HCC progression.

### Forced expression of miR-4651 suppressed cell growth and facilitated apoptosis in HCC cell lines

**Figure 2:**
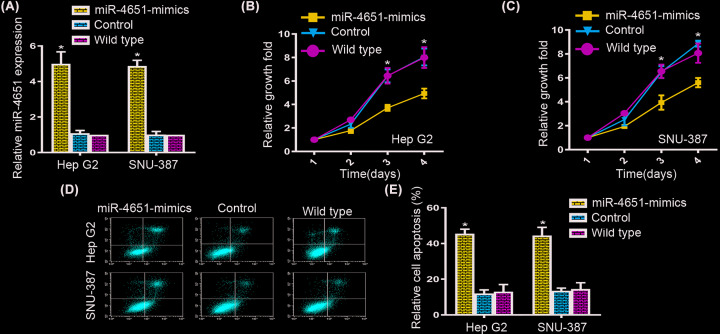
miR-4651 ectopic expression repressed cell proliferation and elevated apoptosis in HCC cell lines (**A**) The expression of miR-4651 was increased in both HepG2 and SNU-387 cells compared with controls and wildtypes after transfection. (**B** and **C**) Cell proliferation assay displayed a inhibition of the growth of HepG2 and SNU-387 cells after miR-4651 transfection compared with the controls and wildtypes. (**D** and **E**) Flow cytometry assay showed the apoptosis rate in HepG2 and SNU-387 cells. The numbers of apoptotic cells were increased compared with the controls and wildtypes in both HepG2 and SNU-387 cells. * in represented the significantly change between miR-4651-mimics and control or wild-type.

According to the transcription level of miR-4651 was decreased in HCC cells, forced expression of miR-4651 was performed to examine the role of miR-4651 in HCC cells. As qRT-PCR analysis shown, the mRNA expression level of miR-4651 was notably elevated in both HepG2 and SNU-387 cells for approximately 5-fold compared with negative controls and wildtypes after transfection (Figure 2A). Then, to figure out the function of miR-4651 in HCC cell growth, we found a dramatically inhibition of the growth of HepG2 cells after miR-4651 transfection compared with the controls and wild-types (Figure 2B). And the same outcomes were observed in SNU-387 cells after miR-4651 transfection (Figure 2C). To inspect the function of miR-4651 in HCC apoptosis, we utilized flow cytometry to calculate the apoptotic cells in both HepG2 and SNU-387 cells, and found ectopic expression of miR-4651 promoted apoptosis in both cell lines (Figure 2D,E). Taken together, our results manifested miR-4651 could suppressed cell growth and enhanced apoptosis in HCC cells.

### MiR-4651 directly interacted with FOXP4 gene and functionally regulated its expression

**Figure 3:**
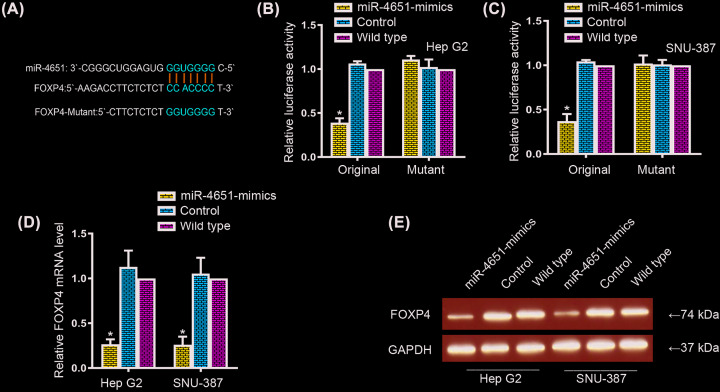
MiR-4651 directly bound to FOXP4 gene and functionally regulated its expression (**A**) Predicted binding sites of miR-4651 in FOXP4 gene and its corresponding mutated form. (**B** and **C**) Binding analysis of original and muted FOXP4 with miR-4651, control and wild-type by luciferase reporter gene assay in HepG2 and SNU-387 cells. (**D** and **E**) The transcriptional and translational levels of FOXP4 were detected after miR-4651 overexpression by using qRT-PCR and Western Blot assays. * in represented the significantly change between miR-4651-mimics and control or wildtype.

To explore the mechanism underlying miR-4651 regulated HCC cell proliferation and apoptosis, we used miRBD to predict the candidate targets of miR-4651, and found a complementary site of miR-4651 in forkhead box P4 (FOXP4). To certify whether FOXP4 is a putative downstream target of miR-4651, we constructed FOXP4 and FOXP4-mutant with mutant binding site of miR-4651 into pGL3 vector with luciferase reporter gene, respectively (Figure 3A). Then, the two plasmids were separately co-transfected with miR-4651 mimics or negative controls into HepG2 and SNU-387 cells. Luciferase assay showed the relative luciferase activity in cells co-transfected with FOXP4 luciferase reporter and miR-4651 mimics was significantly decreased approximately 50% compared with the controls or wild-types; while cells were co-transfected with FOXP4-mutant luciferase plasmid and miR-4651 mimics, the relative luciferase activity was no obviously changes compared with the controls or wildtypes in both HCC cell lines (Figure 3B,C). The outcomes intensively indicated miR-4651 might control the expression of FOXP4 through the putative binding site. Moreover, we also detected the transcriptional and translational levels of FOXP4 after transfection by using qRT-PCR and Western Blot assays. The transcriptional level of FOXP4 was dramatically down-regulated in miR-4651 mimics transfected cells compared with the controls or wild-types in both HepG2 and SNU-387 cells (Figure 3D). In accordance with this, we confirmed the reduction of translational level of FOXP4 in miR-4651 mimics transfected cells in both HepG2 and SNU-387 cells (Figure 3E). In summary, miR-4651 directly interacted with the binding site of FOXP4 and regulated its expression.

### Expression of FOXP4 had an up-regulation in HCC cells

**Figure 4:**
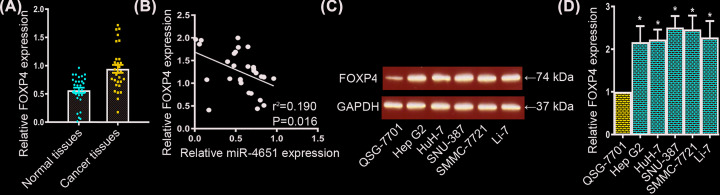
Up-regulation of FOXP4 in HCC tissues and cell lines (**A**) qRT-PCR assay displayed the transcriptional level of FOXP4 was increased in HCC tissues compared with normal liver tissues. (**B**) A negative correlation between the expressions of miR-4651 and FOPX4 in HCC tissues. (**C** and **D**) Both translational and transcriptional levels of FOXP4 were up-regulated in HCC cell lines compared with QSG-7701 using Western Blot and qRT-PCR assays. * in represented the significantly change between HCC cell lines and QSG-7701 cell line.

Above data have revealed that the transcription level of miR-4651 was noticeably decreased in HCC cells, and miR-4651 could directly repressed the expression of FOXP4. Thus, we further explore the mRNA level of FOXP4 in HCC cells. We measured the transcriptional level of FOXP4 using qRT-PCR assay and found FOXP4 was expressed in HCC tissues with a higher level compared with normal liver tissues (Figure 4A). And then, we observed a negative correlation between the expressions of miR-4651 and FOPX4 in HCC tissues (Figure 4B). Meanwhile, we tested the transcriptional level of FOXP4 in HCC cell lines. Consistent with FOXP4 expression in HCC tissues, we authenticated the up-regulation of FOXP4 in both translational and transcriptional levels in HCC cell lines utilizing Western Blot and qRT-PCR assays, respectively (Figure 4C,D).

### MiR-4651 correlated with FOXP4 regulated cell growth and apoptosis in HCC cells

**Figure 5:**
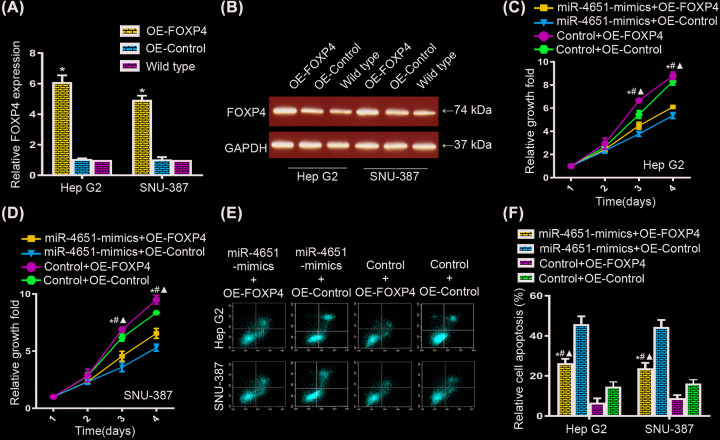
Ectopic expression of FOXP4 promoted HCC cell proliferation and inhibited apoptosis (**A** and **B**) The overexpression of FOXP4 in HepG2 and SNU-387 cells after transfection by qRT-PCR and Western Blot assay. (**C** and **D**) Cell proliferation assay for FOXP4 overexpression correlated with miR-4651in HepG2 and SUN-387 cells compared with OE-controls. The promotion of HepG2 and SUN-387 cell proliferation was remission after miR-4651 and OE-FOXP4 co-transfection compared with OE-FOXP4 transfected cells. (**E** and **F**) The survival of HCC cells was measured by flow cytometry analysis. FOXP4 overexpression co-transfected with miR-4651 led to a promotion of apoptosis in HepG2 and SUN-387 cells compared with OE-FOXP4 cells. * in panel (A) represented the significantly change between OE-FOXP4 and control or wild-type. * in panels (C, D and F) represented the significantly change between mir4651-mimics+OEFOXP4 and control+OE-FOXP4. # represented the significantly change between mir4651-mimics+OE-control and control+OE-control. ▲represented the significantly change between mir4651-mimics+OEFOXP4 and mir4651-mimics+OEcontrol.

To elucidate the interaction between miR-4651 and FOXP4 clearly, we constructed FOXP4 overexpression plasmid (OE-FOXP4) and corresponding negative overexpression control (OE-control) for further research. First, we certified the overexpression of FOXP4 in HepG2 and SNU-387 cells after transfection by qRT-PCR and Western Blot assay (Figure 5A,B). Then, cell proliferation assay showed FOXP4 overexpression led to a promotion in growth of HepG2 and SUN-387 cells compared with OE-controls; while the promotion of HepG2 and SUN-387 cell proliferation were notably remission after miR-4651 and OE-FOXP4 co-transfection compared with OE-FOXP4 transfected cells (Figure 5C,D). Compared with miR-4651 transfected cells, the growths of HepG2 and SUN-387 cells were remarkably promoted in cells with miR-4651 and OE-FOXP4 co-transfection (Figure 5C,D). These data indicated miR-4651 repressed cell proliferation in HCC cells by interacting with FOXP4. For HCC apoptosis, flow cytometry analysis was utlized to measure the survival of HCC cells (Figure 5E). FOXP4 overexpression resulted in a lower death in two HCC cell lines; while co-transfected with miR-4651 and OE-FOXP4, there was a promotion of apoptosis in HepG2 and SUN-387 cells compared with OE-FOXP4 cells (Figure 5F). In conclusion, our studies evidenced a role of miR-4651 in regulating the cell growth and apoptosis in HCC tissues and cell lines through directly interacting with its target gene, FOXP4. Therefore, miR-4651/FOXP4 pathway might be a potential target in therapeutic application of HCC patient treatment.

## Discussion

Hepatocellular carcinoma (HCC), one of the malignant tumors, ranks fifth in morbidity and third in mortality all over the world [2,10,4]. To date, the techniques in the treatment of HCC have been well optimized to reduce the survival rate of HCC patients, but produce very little effects clinically [8]. Despite numerous hypotheses were raised to elucidate molecular mechanism underlying HCC progression, we still haven’t found effective therapies in HCC treatment. Thus, understanding the molecular mechanism of HCC development is fundamental need for diagnostic and therapeutic application of HCC treatment.

Currently, miRNAs have been evidenced to participate in various tumorigenesis, including HCC [12,24]. MiRNAs serve as tumor repressors, and their down-regulation result in a promotion role in tumorigenesis [22,12,24]. Previous studies demonstrate that miR-4651 served as a potential biomarker for prognosis of HCC patients [23], suggesting a crucial role of miR-4651 during HCC progression. In our research, we figured out the expression of miR-4651 was notably decreased in HCC tissues compared with the matched normal liver tissues using qRT-PCR assay. Then, Kaplan–Meier survival curve displayed that patients with low 50% miR-4651 expression suffered worse HCC prognosis compared with high 50% miR-4651 expression. Meanwhile, down-regulation of miR-4651 was detected in HCC cell lines, including HepG2, HuH-7, SNU-387, SMMC-7721 and Li-7 cells compared with the normal cell lines, QSG-7701 cells.

Commonly, miRNAs, act as tumor suppressors, have been proved to inhibit cell proliferation and promote apoptosis. We supposed miR-4651 may repressed cell growth and elevated apoptosis in HCC cells. To verify this point, we perform cell proliferation assay and flow cytometric analysis to test the cell proliferation and apoptosis of HCC cells. Indeed, we found an inhibition in cell growth and promotion in apoptosis in HepG2 and SUN-387 cells after miR-4651 overexpression. These data strongly demonstrated a tumor suppressor role of miR-4651 in regulating the cell growth and apoptosis of HCC cells.

Previous studies manifest miRNAs could functionally target oncogenes or tumor suppressor genes to control tumorgenesis [22,12]. Here, we used miRBD to forecast the potential binding sites of miR-4651 in oncogenes or tumor suppressor genes. Fortunately, we discovered a complementary site of miR-4651 in FOXP4 gene. FOXP4, a member of forkhead box family, is expressed in various organizations, and relate to diverse carcinoma progression [1, 11, 17, 18, 19, 21]. Herein, dual luciferase reporter assay, qRT-PCR and Western blot assay were conducted to confirm the miR-4651 directly targeted FOXP4 gene. Moreover, we also investigated the expression levels of miR-4651 and FOXP4 in both HCC tissues and HCC cell lines, and found a negative correlation between miR-4651 and FOPX4 expressions. Furthermore, forced expression of FOXP4 correlated with miR-4651 sufficiently demonstrated a miR-4651/FOXP4 axis in HCC progression.

## Conclusion

The function of miR-4651 in HCC development was fully elucidated, and we identified a novel pathway, miR-4651/FOXP4 axis, in HCC development, which could be a clinical therapeutic target for HCC patients.

## Abbreviations

CCK-8: Cell Counting Kit-8
FOXP4: forkead box P4
HCC: hepatocellular carcinoma
miRNA: microRNA
miR-4651: microRNA-4651
